# Ongoing haemolytic uraemic syndrome (HUS) outbreak caused by sorbitol-fermenting (SF) Shiga toxin-producing *Escherichia coli* (STEC) O157, Germany, December 2016 to May 2017

**DOI:** 10.2807/1560-7917.ES.2017.22.21.30541

**Published:** 2017-05-25

**Authors:** Sabine Vygen-Bonnet, Bettina Rosner, Hendrik Wilking, Angelika Fruth, Rita Prager, Annelene Kossow, Christina Lang, Sandra Simon, Juliane Seidel, Mirko Faber, Anika Schielke, Kai Michaelis, Alexandra Holzer, Rolf Kamphausen, Daniela Kalhöfer, Sebastian Thole, Alexander Mellmann, Antje Flieger, Klaus Stark

**Affiliations:** 1 Robert Koch-Institute, Department of Infectious Diseases Epidemiology, Berlin, Germany; 2 Robert Koch-Institute, Department of Infectious Diseases, National Reference Centre for Salmonella and other Bacterial Enteric Pathogens, Wernigerode, Germany; 3 University Hospital of Münster, Consultant Laboratory for HUS, Münster, Germany; 4 Postgraduate Training for Applied Epidemiology, Robert Koch Institute, Berlin, Germany; 5 European Programme for Intervention Epidemiology Training (EPIET), European Centre for Disease Prevention and Control (ECDC), Stockholm, Sweden; 6 Ministry of Climate Protection, Environment, Agriculture, Nature Conservation and Consumer Protection of the state of North Rhine-Westphalia, Düsseldorf, Germany; 7 North Rhine-Westphalia, Centre for Health, Department of Infectiology and Hygiene, Münster, Germany

**Keywords:** haemolytic uraemic syndrome, HUS, Shiga toxin-producing E. coli - STEC, outbreak, food-borne infections

## Abstract

We report an ongoing, protracted and geographically dispersed outbreak of haemolytic uraemic syndrome (HUS) and gastroenteritis in Germany, involving 30 cases since December 2016. The outbreak was caused by the sorbitol-fermenting immotile variant of Shiga toxin-producing (STEC) *Escherichia coli* O157. Molecular typing revealed close relatedness between isolates from 14 cases. One HUS patient died. Results of a case–control study suggest packaged minced meat as the most likely food vehicle. Food safety investigations are ongoing.

In February 2017, five cases of haemolytic-uremic syndrome (HUS) were notified in Germany with onset of illness in week 5, 2017, which constituted a marked increase compared with the mean in the same week of the previous 5 years (mean: 0.6; range: 0–2 cases;). In parallel, the consultant laboratory (CL) for HUS at the University Hospital of Münster detected Shiga toxin 2-producing (*stx2*) sorbitol-fermenting (SF) *Escherichia coli* (STEC) O157:H^-^ isolates in four HUS patients with disease onset between December 2016 and February 2017. We initiated an outbreak investigation to identify the cause of the outbreak, in order to control it.

## Epidemiological investigation

**Figure 1:**
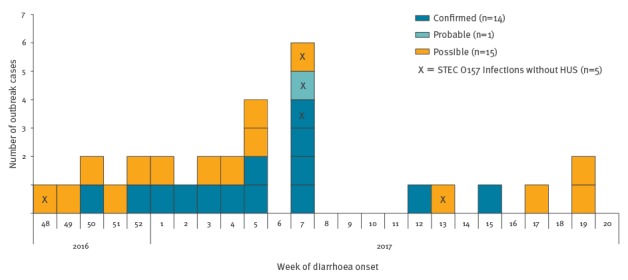
Ongoing haemolytic uraemic syndrome and gastroenteritis outbreak caused by sorbitol-fermenting Shiga toxin-producing *Escherichia coli* O157, Germany, December 2016–May 2017 (n = 30 outbreak cases)

HUS: haemolytic uremic syndrome; STEC: Shiga toxin-producing *Escherichia coli*.

SF *E. coli* O157:H^-^, *stx1*-gene negative, *stx2*-gene positive, *eae-*gene positive was identified as the outbreak strain. As at 22 May 2017, 30 cases, including one family cluster (n = 4), meet the outbreak case definition (Box): 14 confirmed, one probable and 15 possible cases have been reported. The outbreak is ongoing; the most recent symptom onset for a confirmed case was 13 April 2017 (Figure 1).

Box **Case definition, haemolytic uraemic syndrome outbreak caused by sorbitol-fermenting Shiga toxin-producing *Escherichia coli* O157, Germany, December 2016–May 2017**

Outbreak cases were defined as cases of HUS^a^ or STEC infection^b^ with symptom onset starting on 1 December 2016, and residency in Germany.

Confirmed cases were defined as:

• cases of HUS or with STEC infection, with laboratory confirmation of the outbreak strain via WGS base typing or PFGE: SF *Escherichia coli* O157:H-; *stx1*-gene negative, *stx2*-gene positive, *eae*-gene positive.

Probable cases were defined as:

• cases of HUS with laboratory confirmation of SF STEC O157;

• cases of STEC infection with laboratory confirmation of SF O157;

• cases of HUS, STEC infection or bloody diarrhoea with an epidemiological link to a confirmed or probable case.

Possible cases were defined as:

• cases of HUS without any pathogen verification, or *E. coli* without confirmation of SF characteristic, or with confirmation of *E. coli* serogroup O157, excluding individuals with a laboratory test result conflicting with any characteristic of the outbreak strain, e.g. other serogroup.

• cases of STEC infection with *E. coli* serogroup O157 laboratory confirmation;

• cases of HUS, STEC infection or bloody diarrhoea with an epidemiological link to a possible case.

HUS: haemolytic uremic syndrome; PGFE: pulsed-field gel electrophoresis; SF: sorbitol fermenting; STEC: Shiga-toxin producing *Escherichia coli*; WGS: whole genome sequence.
^a^ HUS is defined as at least two of the following three criteria: haemolytic anaemia, thrombocytopenia ≤ 15.000 cells/mm^3^ or renal impairment; or diagnosis of acute enteropathic HUS by a clinician, or death caused by HUS.
^b^ STEC infection clinically defined.

**Figure 2:**
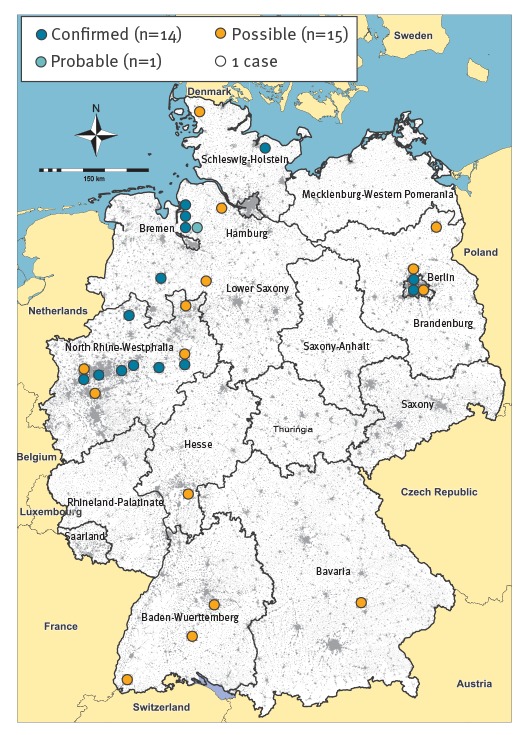
Geographic distribution of cases, ongoing outbreak of haemolytic uraemic syndrome and gastroenteritis caused by sorbitol-fermenting Shiga toxin-producing *Escherichia coli* O157, Germany, December 2016–May 2017 (n = 30 outbreak cases)

The 14 confirmed cases resided in the north-west of Germany and Berlin (Figure 2).

The grey areas indicate densely populated regions.

The mean age of the confirmed cases was 8.5 years (range: 1–36), half of them were male, 13 developed HUS and needed intensive care treatment; one patient died. None of the cases had a relevant pre-existing illness. The probable case, a middle-aged adult, a STEC enteritis case, was a close family member of three confirmed cases. Twelve of the 15 possible cases developed HUS and three had gastroenteritis; the mean age of the possible cases was 26 years (range: 0–83) and seven were male.

## Explorative interviews

We interviewed 11 of the confirmed cases or their parents using a standardised trawling questionnaire containing questions on clinical symptoms, travel history, animal contacts, farm visits, other leisure activities and food consumption during 10 days before symptom onset.

During 11 explorative interviews, we identified a number of frequently named food items, e.g. minced meat (beef and pork mixed), hot dog-style (Vienna) sausages, as well as several commercially available yoghurts or puddings. Cases and parents also reported having frequently shopped at supermarket chain X. They reported no common place of exposure. None of the cases had travelled in Germany or abroad. Few had had contact with a dog or cat (n = 4), one case had had contact with a donkey, a horse, a sheep, and a goat. One had consumed unpasteurised milk. None reported a farm visit or contact with cattle. This supported our initial hypothesis of a food-borne outbreak, which we had suspected based on biological plausibility and the wide geographical distribution of cases.

## Case–control study

Following the interviews we conducted a case–control study to investigate the association of illness with food items named frequently in the explorative interviews. For each confirmed case under 13 years of age, we recruited three to six children reported to the local health authorities with another notifiable disease (e.g. influenza), who lived in the same or a neighbouring district as the outbreak case, and belonged to the same age group (1–4; 5–9; 10–12 years). Parents of cases and controls were interviewed by telephone after they had given informed consent. We used a short standardised questionnaire which covered the consumption of the above mentioned food items in the 10 days before symptom onset with details such as brand names, packaging, shopping places, etc.

We analysed the data using MS Excel and Stata 14.1 (Stata Corporation, Texas, United States), compared cases and controls by Chi-squared and Student t-test and estimated matched odds ratios (OR) for consumed food items and supermarkets using the Mantel-Haenszel test.

We included nine confirmed cases and 35 individually matched controls in the analysis. Cases and controls did not differ by sex and age; there was no association between being a case and having consumed different types of desserts (data not shown). Cases had eaten minced meat (beef and pork mixed) significantly more frequently than controls (Table). All cases (6/6) with the respective information reported this exposure. The parents of the cases prepared minced meat at home significantly more frequently than parents of the controls and they were significantly more likely to have purchased the minced meat at supermarket chain X (OR: 14.1; 95% confidence interval (CI): 1.2–174.9). However, only three of eight cases were explained by this exposure and the CI was wide. Shopping mostly or exclusively at supermarket chain X was associated with a threefold odds for being a case (OR: 3.0; 95% CI: 0.8–11.4).

**Table:** Association between being a case and having consumed certain food items, ongoing haemolytic uraemic syndrome and gastroenteritis outbreak caused by sorbitol-fermenting Shiga toxin-producing *Escherichia coli* O157, Germany, December 2016–May 2017 (n = 9 cases;  n =  35 controls)

Variable	Exposed cases		Exposed controls		OR	95% CI ^ ^	p value
**Consumption of**	**n**	**%**	**n**	**%**			
**Heated minced meat**	**6/7**	**86**	**19/33**	**58**	**5.5**	**0.6–52.9**	**0.096**
Heated mixed minced meat (beef and pork)	**6/6**	**100**	**9/23**	40	NC	NA	**0.015**
Minced meat bought from supermarket chain X	3/8	38	1/33	3	**14.1**	**1.2–174.9**	**0.007**
Minced meat bought from supermarket chain Y	2/7	29	6/35	17	2.0	0.4–10.4	0.424
Raw minced meat	1/8	13	5/34	15	2.1	0.09–49.2	0.637
Vienna sausages	6/8	75	8/34	35	6.3	0.6–63.6	0.073
Vienna sausages bought from supermarket chain X	4/6	67	5/12	42	2.3	0.2–28.3	0.493
Vienna sausages bought from supermarket chain Y	1/6	17	3/12	25	0.5	0.02–14.9	0.683
Preparation of dish with minced meat in the household (at least once a week versus less than once a week)	7/9	78	11/35	31	**8.6**	**1.2–60.6**	**0.009**
Shopping at supermarket chain X (always or mostly)	5/8	63	9/35	26	3.0	0.8–11.4	0.087

CI: Confidence interval; NA: not applicable; NC: not calculated; OR: odds ratio.

Significant results are displayed in bold.

## Microbiological investigation

The microbiological investigations at the National Reference Centre for *Salmonella* and other Bacterial Enteric Pathogens at the Robert Koch Institute (RKI, national public health institute) and the CL for HUS included serotyping, testing for Shiga toxins, other phenotypic and genotypic markers and molecular subtyping (PFGE and WGS). We applied a threshold of > 10 alleles difference to securely exclude isolates from the outbreak [1]. Raw reads of the index case are available for download at http://www.ebi.ac.uk/ena under the study accession number PRJEB20962.

Molecular typing revealed close relatedness between isolates from 14 confirmed cases. The probable case had a PCR-positive stool sample sharing outbreak specific markers but culturing from stool was unsuccessful. Of the possible cases, all three STEC enteritis and four of the twelve HUS cases were positive for STEC O157, without any further typing. For the remaining eight HUS cases, isolates were not available.

## Food safety investigations

State food safety authorities conducted inspections and sampling at minced meat producing plants supplying supermarket chain X and others. All results of official samples on production sites, retail level and from regional monitoring programmes have been negative for SF STEC O157 so far. Trace-back investigations starting from locations where cases had purchased minced meat or hot dog-style (Vienna) sausages are ongoing.

## Discussion

We report an ongoing, protracted and geographically dispersed outbreak of HUS and gastroenteritis in Germany caused by SF STEC O157:H^-^. The supra-regional occurrence of cases makes a food item the most likely vehicle of transmission. Based on the investigations to date, we suspect packaged minced meat (beef and pork mixed) sold at one or several supermarket chains, as the most likely source. Other food items purchased at supermarket chains, in particular pre-packaged hot-dog style (Vienna) sausages, cannot be excluded as vehicle at this moment in time, but appear to be less plausible mainly for two reasons: less cases can be explained by this exposure compared to minced meat and the sausages are heated during the production process and therefore contamination appears less likely.

SF STEC O157:H^-^ was first described in Germany in 1988 [2] and subsequently caused several outbreaks in Germany [3-5] and elsewhere in Europe [6,7]. SF STEC O157 is characterised by a high pathogenicity [8,9]. One of the patients in the present outbreak died, and all 10 HUS cases whose parents were interviewed needed intensive care and dialysis. Few secondary gastroenteritis cases among family members were observed. Similarly to previously described outbreaks caused by SF STEC O157, this outbreak spanned several months and cases were geographically dispersed [4,5].

Little is known about the SF STEC O157 reservoir. In previous investigations, the pathogen was found in cattle and reindeer [10-12]. In other outbreaks, SF STEC O157:H^-^ infections were epidemiologically linked to the consumption of mortadella (Italian-style sausage) and smoked pork paté [5] or to visiting one particular playground [3]. SF STEC O157 was isolated from minced beef products in an outbreak involving 18 HUS cases in France in 2011 [6]. In an outbreak associated with a recreational farm visit in Finland in 2012, unpasteurised milk was the most likely vehicle and isolates from patients’ stool samples, cattle and the farm environment were identical [7].

At this point, investigations focus on minced meat as outbreak vehicle, although the available evidence is weak. The source of the contamination with SF STEC O157 is so far unknown. For minced meat, contaminations at the farm, slaughterhouse or subsequent production steps, are all possible options. It is thus conceivable that several retail outlets may be concerned.

One limitation in the investigation of this outbreak is that the number of interviewed confirmed cases is small and therefore, results of the case–control study should be interpreted with caution. For a number of HUS cases, no isolate was available and there was no opportunity of laboratory confirmation. In order to identify outbreak cases and allocate them appropriately, diagnostic laboratories and public health services are requested to send any STEC isolate from HUS patients to a typing centre. The use of genomic methods is desirable as routine for characterising all isolates. 

A further limitation is that for a substantial proportion of cases, typing and interviews could not be conducted at all, or only weeks after symptom onset. Physicians, diagnostic laboratories, including national reference and consultant laboratories, public health and veterinary authorities at local, state and federal level, should be aware that timely actions are crucial for early detection of the vehicle and source.

Several previous investigations into outbreaks with SF STEC O157 have been unsuccessful in identifying the source, despite intensive efforts of health and food safety authorities [3-5]. This is the largest outbreak of SF STEC O157 in Germany since 2002 [4] and it has lasted for more than 4 months already. We assume that the source of infection may still be active and further cases may still occur. According to the feedback obtained via the Epidemic Intelligence Information System (EPIS) of the European Centre for Disease Prevention and Control (ECDC), the German outbreak strain has not been detected in other European countries. In Germany, continued epidemiological investigations, sampling of food isolates and trace-back of food items are needed and ongoing to identify the cause of the outbreak.
